# Chemical effects on the dynamics of organic molecules irradiated with high intensity x rays

**DOI:** 10.1063/4.0000166

**Published:** 2022-10-31

**Authors:** Sourav Banerjee, Zoltan Jurek, Malik Muhammad Abdullah, Robin Santra

**Affiliations:** 1 Center for Free-Electron Laser Science (CFEL), Deutsches Elektronen-Synchrotron (DESY), Notkestr. 85, 22607 Hamburg, Germany; 2 The Hamburg Centre for Ultrafast Imaging, Luruper Chaussee 149, 22761 Hamburg, Germany; 3 Deutsches Elektronen-Synchrotron (DESY), Notkestr. 85, 22607 Hamburg, Germany; 4 Department of Physics, Universität Hamburg, Notkestr. 9-11, 22607 Hamburg, Germany

## Abstract

The interaction of a high intensity x-ray pulse with matter causes ionization of the constituent atoms through various atomic processes, and the system eventually goes through a complex structural dynamics. Understanding this whole process is important from the perspective of structure determination of molecules using single particle imaging. XMDYN, which is a classical molecular dynamics-Monte Carlo based hybrid approach, has been successful in simulating the dynamical evolution of various systems under intense irradiation over the past years. The present study aims for extending the XMDYN toolkit to treat chemical bonds using the reactive force field. In order to study its impact, a highly intense x-ray pulse was made to interact with the simplest amino acid, glycine. Different model variants were used to highlight the consequences of charge rearrangement and chemical bonds on the time evolution. The charge-rearrangement-enhanced x-ray ionization of molecules effect is also discussed to address the capability of a classical MD based approach, i.e., XMDYN, to capture such a molecular phenomenon.

## I. INTRODUCTION

The generation of highly intense ultrashort pulses using x-ray free electron lasers (XFELs)^1–6^ has revolutionized various fields of science, e.g., studying molecular structures with a spatial resolution of atomic scales and with a temporal resolution of femtoseconds.^7–20^ It is of fundamental importance to understand the interaction of high intensity x rays with atoms and molecules in order to master XFEL-based techniques. High fluence may even push the probability of atomic ionization to saturation, whereas other competing processes occur subsequently, making the initiated dynamics highly complex. With rapid interest in single particle imaging^21^ for biomolecules and the advancement of peak brightness^22^ as well as focusing capabilities for the x-ray pulses,^23^ it is not far to reach, even in the hard x-ray regime, a situation where multiple photons are absorbed by individual atoms. The interaction with an intense x-ray pulse is mainly governed by sequential multi-photon multiple-ionization dynamics. The inner shell electrons are knocked out first due to photoionization, and then the relaxation of the electronic configuration takes place via Auger decay or fluorescence. In both cases, the core hole is refilled by a valence electron, whereas another valence electron (Auger electron) or a photon is ejected, carrying the excess energy away. These processes repeat sequentially in the high intensity regime. Moreover, in spatially extended matter, the environment influences the dynamics further through collisional ionization and recombination events.

The mixed Monte Carlo-molecular dynamics (MD) based computational tool XMDYN^24^ has been applied over the past several years to deal with situations where molecules,^25,26^ clusters,^27^ and nanocrystals^28^ were irradiated with high intensity x-ray pulses. The agreement of the simulation results using XMDYN with experimental observations has already established this hybrid approach to be a promising framework to investigate the dynamical evolution of a molecular system under highly intense x-ray pulses. Nonetheless, XMDYN is still missing a general approach to treat chemical bonds between the atoms as it considered either system specific bonds in a few earlier studies^25,27,29^ or the chemical bonds were just neglected as the highly intense x-ray pulses are expected to break the bonds at a very early stage of exposure. However, specific applications as well as practical aspects of realistic XFEL experiments (e.g., the consequences of the laterally inhomogeneous spatial intensity profile^30^) demand a proper theoretical description at moderate fluence, too. This leads to the question whether the MD based scheme can be further developed to deal with a situation where a moderate fluence of incident x rays ionizes atoms to an extent that the molecule is not fully fragmented, and chemical bonds still play a role in the dynamical evolution of the system.

Following accurately the changes of the chemical bonds in an electronically highly excited, ionized system is very challenging. One strategy, at least to address a limiting case of the dynamics with maximized bond effects, is the application of the bondings valid for neutral systems, irrespective of the charge state of the atoms. In order to do so, the reactive force field (ReaxFF) method^31^ has been applied here because its inter-atomic potentials describe reactive events, vibrationally highly excited states and even fragmentation of a neutral system in its ground electronic state. With its newest extension, the code aims for capturing the effect of chemical bonds via interfacing to an implementation of the reactive force field method, the ReaxFF code.^32–34^ This latest development in XMDYN appears in the focus of the current paper.

When a molecule is irradiated with x rays, the interaction of the radiation is significantly stronger with heavy atomic constituents compared to the lighter ones. At low fluences, where the probability of photoionization during the pulse is much less than one, the similarity of ionization dynamics for a molecule and for its atoms isolated from each other has been found justified. However, the situation is very different at higher fluence. As a heavy atom within the molecule is ionized most strongly due to photoabsorption, it draws electrons from lighter neighbors. These electrons are then available for further inner shell ionization processes on the heavy atom, resulting in a significant increase in the total molecular charge, compared to the independent atom scenario. This effect is known as charge-rearrangement-enhanced x-ray ionization of molecules (CREXIM). CREXIM was first reported in a theoretical study using the quantum mechanics based code XMOLECULE^35^ and was observed experimentally^18^ as well. We take the opportunity in this paper to demonstrate that XMDYN, which uses a hybrid modeling approach, can capture the CREXIM effect. CREXIM has not been reported in any earlier studies done with XMDYN.

In this paper, the dynamics of glycine molecules induced by high intensity x-ray irradiation is studied using XMDYN in conjunction with ReaxFF. The calculations are performed at three different fluences, i.e., 10^13^, 10^14^, and 10^15^ photons/*μ*m^2^, and at a photon energy of 12 keV. The fluences chosen here cover a range of scenarios from the situation when a single atom may not even absorb, on average, a single photon (e.g., at the lowest fluence, 10^13^ photons/*μ*m^2^, considered), to the one when it absorbs multiple photons at the highest fluence (10^15^ photons/*μ*m^2^). The lowest value is realistically achievable,^21^ while the intermediate one is a design goal for XFELs.^36^ The highest fluence is out of reach at present with the photon energy used here. However, we note that by reducing the photon energy, the photoionization cross section increases nonlinearly, effectively leading to similar photoionization probabilities during a single pulse. Furthermore, glycine is a prototypical choice not only because of its biological importance as an amino acid but also for being a small molecule. The molecule, being small, is not affected by environmental effects like collisional ionization or recombination, which, otherwise, would hinder the focus of looking into changes appearing solely through bonds. Therefore, with this rational choice, we perform calculations using three different versions of XMDYN:

(1) Basic (reference) XMDYN framework, i.e., the framework described in Ref. 24 (XMDYN-REF).
(2) XMDYN with charge transfer (XMDYN-CT).
(3) XMDYN with charge transfer and with the ReaxFF extension (XMDYN-CT-FF).

This paper is organized as follows. Section II on methodology describes the three different XMDYN schemes briefly. In Sec. III, in order to unfold the effects and importance of the charge transfer and chemical bonds, we discuss the fragmentation behavior and give insight into the temporal behavior of the molecular as well as the atomic partial charges, together with the time dependent atomic displacements (the latter quantity being in the focus of x-ray imaging experiments). We provide concluding remarks in Sec. IV.

## II. METHODOLOGY

### A. Basic XMDYN framework

XMDYN is a computational tool designed to simulate the dynamics of matter irradiated with high-intensity x rays. Neutral atoms, atomic ions, and ionized electrons are considered as classical particles in the XMDYN framework. The real space dynamics of these particles is obtained by applying the classical MD technique. Regarding the bound electrons, XMDYN uses a simplified electronic structure description by disregarding the molecular nature of orbitals, and, instead, assuming orbitals of isolated atoms. The electronic configuration of each atom is accounted for using orbital occupation numbers. XATOM^24^ calculates the rates and cross sections for photo-induced processes such as photoionization, Auger decay, and fluorescence for any electronic configuration that occurs during the pulse exposure (and afterward). XMDYN loads the data produced by XATOM on the fly into its Monte Carlo algorithm to track the stochastic changes in the electronic configurations. Secondary ionization and recombination processes are also included in this framework. The algorithm identifying secondary ionization events is parametrized by the binary-encounter-Bethe (BEB) cross section^37^ supplied from XATOM and is based purely on the real-space dynamics of the particles (i.e., without random number generation). Recombination of classical electrons into quantum orbitals is allowed to take place when an electron revolves around an ion for certain periods. However, the effect due to secondary ionization and recombination processes is negligible in the case of the studied molecule.

This basic framework of XMDYN, serving also as a reference in the current study, will be referred to as XMDYN-REF in the following.

### B. XMDYN with charge transfer

During the time evolution of a system charge imbalance may occur between neighboring atoms due to their different temporal ionization histories. The phenomenon can be even more significant between atoms of different elements. Charge imbalance can lead to charge transfer between atoms, which, within the XMDYN framework, can be captured by changing the corresponding orbital occupation numbers and charge state of the atoms involved: the occupation number of the donor orbital of the electron donor atom (D) is decreased by one, while the occupation number of the acceptor orbital of the electron acceptor atom (A) is increased by one at the same time.

An extended version of XMDYN employed a charge transfer algorithm based on the classical over the barrier (OTB) model^38^ in a few earlier studies.^27,29^ In the simple OTB based approach, an atomic bound electron is considered to be free to move from the donor (D) atom/ion to a neighboring acceptor (A) ion if the Coulomb barrier built up by the field of A, and the ionic core of D is below its orbital energy. The systems investigated in Refs. 27 and 29 consisted of neutral and singly charged atoms with the same atomic numbers, and only valence shell electrons were hopping among the neighboring atoms. In such situations, the charge transfer led effectively to the interchange of electronic configurations of the participating atoms, causing no significant change in the energy contained in the atomic bound electron subsystem. Though a slight energy difference during charge transfer might occur because of the altered classical Coulomb potential, the energy conservation is still preserved via transferring this small amount of energy as kinetic energy to the ion cores.

However, for systems with different atomic species, this kind of approach is inappropriate as large energy differences might occur, leading to unphysically large sudden changes of the atomic kinetic energies. A different approach,^39^ applied also here, is based on the OTB model as well, but disregards any changes of the total energy due to different energy levels involved in the charge transfer and changes due to the sudden alternation of atomic charges. This means that energy is not transferred to the atomic ions, unlike the previous consideration. Within this approach, only electron transfer between energetically closest orbitals is considered, but disallowing the participation of core holes in the process.

Charge hopping between two neighboring atoms remains feasible up to a critical interatomic distance, beyond which the potential barrier becomes so high that the passage of an electron from one atom to another atom becomes forbidden by the OTB condition. Therefore, the charge on each atomic site remains the same beyond this separation distance. We also note that the two directions in which the electron may hop within a two atom (sub-)system are asymmetric if the binding energies of the outermost bound electrons of the two atoms, being in their donor state, are different. Therefore, it might occur in a certain interatomic distance range that although the transfer of an electron from one of the atoms/ions to the other is allowed, the reverse channel is forbidden, and, hence, charge transfer in the corresponding direction stops at that point.

XMDYN-CT, an abbreviation for referring to this version of the simulation framework, i.e., XMDYN including charge transfer, appears in the subsequent discussion.

### C. XMDYN with charge transfer and ReaxFF

A pure quantum mechanical description of chemical phenomena, e.g., of chemical bonds, can lead to extreme computational times with increasing system size. Therefore, as an approximation, the reactive force field (ReaxFF) interatomic potential is used here to describe the bonding between atoms. ReaxFF has been developed as a general force field to capture chemical bonds within wide groups of neutral molecules in their ground electronic state (e.g., the group of proteins) through proper parameterization. As an approximation, we assume that the functional forms of the bonds and their parameters remain the same irrespective of the molecular charge state. This approximation, not allowing for bond softening, overestimates the effect of chemical bonds, and this way such an approach presents a limiting case of modeling in addition to the bondless framework. Of course, the repulsive Coulomb forces among atomic ions do increase with the charge, but they are distinct from bonding effects.

ReaxFF utilizes the concept of bond order to describe how atoms interact with each other. With this, one can obtain a dynamic description of atomic/molecular interactions that is not dependent on predefined reactive sites as in the case of other empirical potentials. In ReaxFF, this is achieved by a detailed parameterization of different properties of each particle and interaction within the system, against *ab initio* calculated and experimental data.

ReaxFF takes care of the bonded as well as the non-bonded interactions. For bonded interactions, the following steps are taken into consideration. (i) Bond order is determined using the positions of the atoms in the system. (ii) Correction of bond order for overcoordination, i.e., correcting carbon interactions at different bond orders. (iii) In ReaxFF, the angle and torsion interactions are also bond order dependent. When an atom breaks a bond and leaves a molecule, the force exerted on it due to angle and torsion with respect to the rest of the molecule weakens smoothly along with the bond order. For non-bonded interactions, (i) the polarization of charges within the molecules is determined; (ii) the Coulomb and Van der Waals forces among all the atom pairs, irrespective of their connectivity, are calculated.

Here, we use the original ReaxFF implementation^34^ interfaced to XMDYN with the generalized protein force-field parameters.^31^ A detailed description of ReaxFF is given in Ref. 40. Simulation results performed using this version of XMDYN are referred to in the following as XMDYN-CT-FF.

## III. RESULTS AND DISCUSSION

**FIG. 1.**
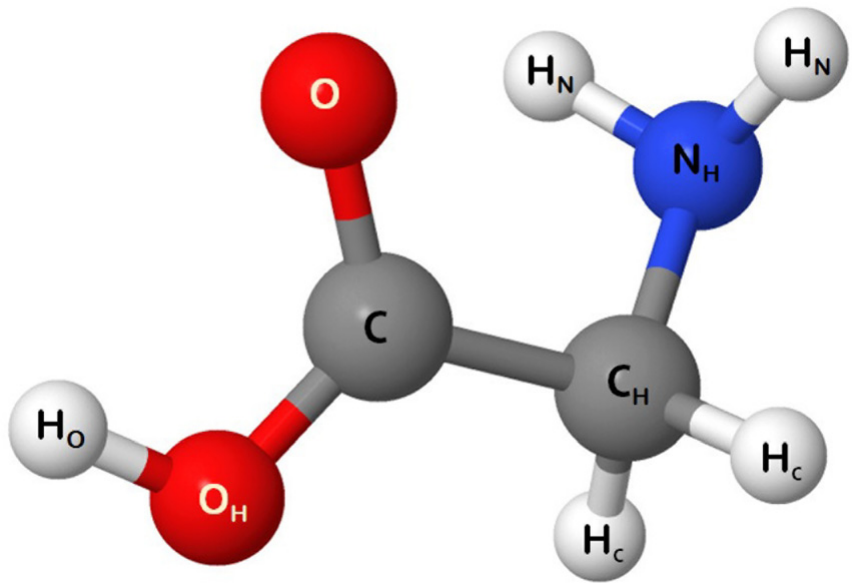
A ball-stick representation of a glycine molecule. The presence (or absence) of lower indices in the labeling indicates the presence (or absence) of connectivity to hydrogen (in the case of C, N, and O) or non-hydrogen (in the case of H) atoms.

With a motive to investigate how different features in the aforementioned schemes manifest themselves in the dynamical evolution of a molecule exposed to a highly intense x-ray beam, we perform simulations on single glycine molecules irradiated with a 12 keV x-ray pulse, having a duration (full width at half maximum) of 10 fs, and at three different fluences, i.e., 10^13^, 10^14^, and 10^15^ photons/*μ*m^2^. Comparison among the results obtained using these three variants of simulation highlights the effects of physical phenomena on the dynamics in different intensity regimes. Glycine, as shown in Fig. 1, is a small molecule with only ten atoms and, hence, beyond atomic processes, exhibits a time evolution affected by chemical phenomena without the interplay of processes emerging in a dense environment inside larger molecules (secondary ionization and recombination). In order to obtain results with sufficiently small statistical error for a reliable analysis, we compute 4000 realizations for 10^13^ photons/*μ*m^2^ and 1000 realizations each for 10^14^ and 10^15^ photons/*μ*m^2^. The dynamics of the molecule is investigated up to 1 ps after irradiation within which all the important processes have taken place, providing a conclusive scenario already. The temporal evolution is obtained using time steps of 1 as.

### A. Fragmentation

The interaction of a high intensity x-ray pulse with a molecule causes ionization of the constituent atoms of the molecule, creating charge on different atomic sites. Depending on the charge created due to ionization and subsequent Auger decay, the atoms exert repelling forces on each other. The Coulomb repulsion may breakup the molecule into fragments. The details of fragmentation depend on the model variant used. In order to get later a better understanding of the molecular properties, we first analyze the fragment relative yields at the final time point of the simulations, using the three modeling schemes considered. Only those trajectories that end with nonzero total charge are considered for this analysis (nonionized trajectories trivially lead to the unfragmented case). A group of atoms is considered to be an already separated fragment when all nearest neighbor distances within the group are less than a predefined threshold length, while the group is separated from the remaining atoms by more than this threshold. In our analysis, we chose this length to be 10 Å.

**FIG. 2.**
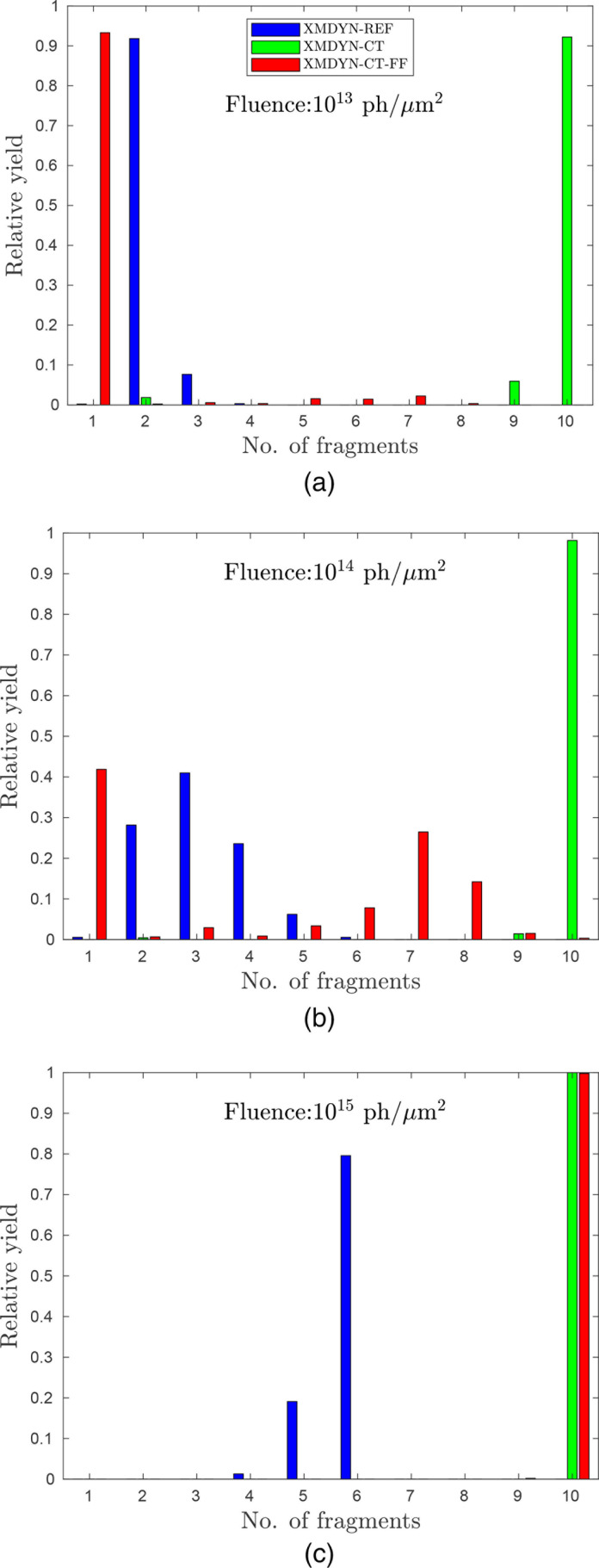
Relative yields of oligomers with different sizes at 1 ps after irradiation, for fluences 10^13^ photons/*μ*m^2^ (a), 10^14^ photons/*μ*m^2^ (b), and 10^15^ photons/*μ*m^2^ (c), using the three model variants described in the text. Only ionized trajectories are considered in the statistics. A nonionized molecule forms, naturally, a single oligomer irrespective of the model applied.

Figure 2 shows the relative yield of oligomer classes containing different numbers of atoms (but irrespective of the fragment charge state), for all three modeling scenarios at the three fluences. The ratio of the number of trajectories with at least a single ionization event is 23%, 92%, and 100% for 10^13^, 10^14^, and 10^15^ photons/*μ*m^2^, respectively. For the fluence of 10^13^ photons/*μ*m^2^, Fig. 2(a) shows that the independent atom consideration of XMDYN-REF provides a high relative yield for trajectories with two final fragments. At such a fluence, the most probable scenario is that only one of the non-hydrogen (non-H) atoms (i.e., C, O, or N) gets ionized. That atom acquires a slight recoil momentum at the ejection of the ionized electron, and this eventually is enough (having zero Coulomb force between any of the atoms and no bonds at all as per XMDYN-REF) to separate the atom from the remaining part of the molecule. Ionization at more atomic sites can lead to more fragments due to the repulsive force. The yields corresponding to these situations (due to the small probability of multiple photoionization) are negligible. The XMDYN-CT scheme (which includes charge transfer) indicates that full fragmentation has the highest yield. This is evident because of the following: (i) typically, the molecule is doubly charged due to the atomic photoionization-Auger cycle; (ii) due to charge transfer, the two charges are separated and appear at two sites; (iii) all the atoms of the molecule acquire charge transiently due to charge transfer; (iv) therefore, they gain momentum due to Coulomb repulsion (v) that eventually, due to the absence of chemical bonds, makes them fly away from each other, resulting in full atomic fragmentation. Very small relative yields corresponding to smaller numbers of fragments also appear, but that happens since the system is analyzed at a finite time (1 ps) and in rare cases such slow atoms appear that some of the nearest neighbor distances reach the threshold distance introduced for the identification of fragmentation only at a later time. If the bonds between the atoms are accounted for, as is done in XMDYN-CT-FF, the atoms are mostly held together by the bonds, as the Coulomb repulsion among the atoms is not strong at low fluence. Therefore, we observe the maximum yield for just one fragment in XMDYN-CT-FF, which indicates that the atoms typically stay together as a molecule.

As the fluence increases to 10^14^ photons/*μ*m^2^ [Fig. 2(b)], ionization becomes more likely, and the higher molecular charge leads to stronger fragmentation. XMDYN-REF shows significant relative yields for larger numbers of fragments as more atomic sites possess nonzero charge. XMDYN-CT shows full fragmentation just as before. Finally, in the case of XMDYN-CT-FF, the Coulomb repulsion between the charged atoms might create enough excitation to break some of the bonds and tear the molecule into fragments. The relative yields in XMDYN-CT-FF indicate that the ensemble of trajectories leads to a mixture of unfragmented and fragmented molecules at the final time point.

When the fluence is as high as 10^15^ photons/*μ*m^2^, all non-H atoms undergo ionization multiple times, which leads to strong Coulomb repulsion among the atoms. In the XMDYN-REF scheme, hydrogens do not move with time as they are still not ionized due to their very small photoionization cross section, whereas the non-H atoms fly apart. Therefore, the five hydrogens counted as one fragment, and the five non-H atoms being ejected result in a total of six fragments [Fig. 2(c)]. The XMDYN-CT scheme again gives rise to full fragmentation. XMDYN-CT-FF also shows full fragmentation as the charge is spread among all (including hydrogen) atoms due to charge transfer and the Coulomb forces highly overwhelm the chemical bonds.

The following discussion of the total molecular charge and the partial charges on the atoms will provide more insight into the dynamical evolution of the system.

### B. Total molecular charge

In the analysis of the x-ray pulse driven dynamics of matter, an important quantity is the overall probability of photoionization,

P=1−exp (−σF), (1)

connected to the product of the cross section of the process (*σ*) and the fluence (*F*) applied.

For the case of σF≪1, the probability can be approximated simply by P∼σF (linear regime). In the case of 1≲ σF, multiple photoionization is highly probable (nonlinear regime). The saturation fluence,^41^ $F_{\mathrm{sat}}=1/\sigma$, is a useful reference quantity in the analysis. The photoionization cross section for the atoms of glycine, namely, for H, C, N, and O, and for the whole molecule is $2.5\times 10^{-19}$, $2.3\times 10^{-15}$, $4.6\times 10^{-15}$, $8.3\times 10^{-15}$, and $2.6\times 10^{-14}$ μm^2^, while the corresponding saturation fluence values are $4.0\times 10^{18}$, $4.4\times 10^{14}$, $2.2\times 10^{14}$, $1.2\times 10^{14}$, and $3.9\times 10^{13}$ photons/ μm^2^, respectively.

**FIG. 3.**
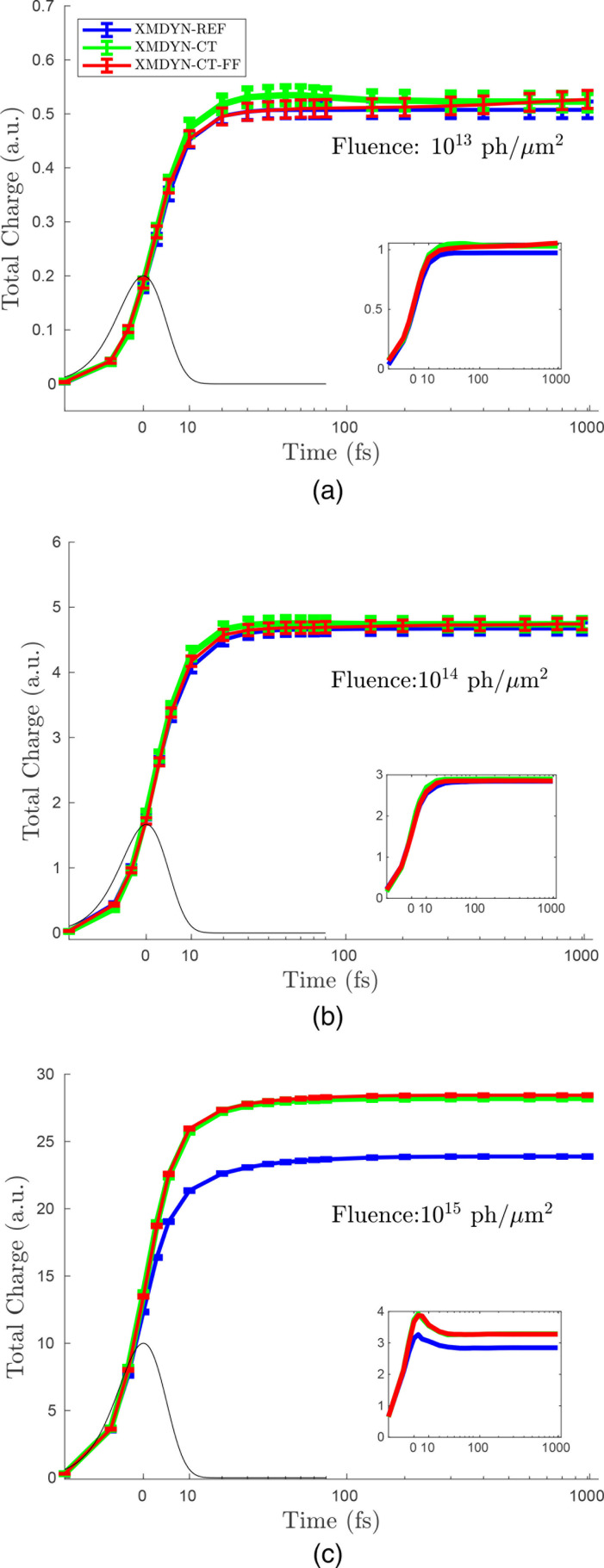
Average total charge of glycine as a function of time obtained using three models (blue for XMDYN-REF, green for XMDYN-CT, and red for XMDYN-CT-FF) for three different fluences: (a) 10^13^ photons/*μ*m^2^, (b) 10^14^ photons/*μ*m^2^, and (c) 10^15^ photons/*μ*m^2^. The error bars show the statistical error. Insets: standard deviation of the total molecular charge as a function of time.

The total charge of the molecule throughout its dynamical evolution, averaged over the simulated trajectories, is depicted in Fig. 3. Each panel represents the results corresponding to one particular fluence, and different colors indicate the results from different simulation schemes. We note that the lowest fluence is well below all saturation fluence values, and the intermediate one is approximately equal to the saturation fluence of the molecule, whereas the highest fluence exceeds the saturation fluence of all non-H atoms. We also note that the probability of directly photoionizing a hydrogen atom is negligible throughout the fluence regime investigated.

Comparing the three panels of Fig. 3, we observe that the average total charge for the lowest fluence [Fig. 3(a)] is very small. We also observe an evident increase in the total charge for all three approaches as we go from 10^13^ to 10^14^ photons/*μ*m^2^ [Fig. 3(b)] and then to 10^15^ photons/*μ*m^2^ [Fig. 3(c)], which is trivial because more photoionization and Auger decay events occur for higher intensities. Furthermore, we notice that the total charge gradually goes up as a function of time. This is obvious for every fluence because photoionization becomes more probable as the number of photons arrived rises, and the Auger decays occur with a sub-ten-femtosecond lifetime after photoionization, leading to a further delay in reaching the final total charge.

Another important observation is that the total charge is always higher for XMDYN-CT and XMDYN-CT-FF than for XMDYN-REF. This difference becomes more significant as the fluence increases. This behavior is due to the CREXIM effect alluded to in Sec. I. In XMDYN-REF, all the atoms are treated independently, and hence, the heavier atoms (O, N, and C) are most likely to get ionized, whereas no ionization takes place from H atoms. On the other hand, the inclusion of charge transfer in XMDYN-CT and XMDYN-CT-FF brings molecular effects into play: there is charge rearrangement as soon as ionization occurs on any non-H atom, and therefore, H atoms also get charged up through charge transfer. As the probability of single photoionization is much smaller than 1 at the lowest fluence, the non-H atoms typically do not acquire (via a second photoionization event) additional charge to share. However, with increasing fluence, all these heavier atoms are more likely to be photoionized several times during the pulse, eventually leading to CREXIM. If all the H atoms in the molecule permanently change to H^+^ at certain stage of the dynamics (which occurs when all H and non-H atoms are charged), then this leads to five extra charges on the molecule compared to the XMDYN-REF case, where H atoms never get ionized. This is exactly what we observe in Fig. 3(c) for the XMDYN-CT and XMDYN-CT-FF models at the highest fluence investigated.

The standard deviation is a useful quantity to represent the trajectory-by-trajectory fluctuation of a quantity. At the lowest fluence, the behavior of the standard deviation of the total charge [inset of Fig. 3(a)] can be understood via introducing a stochastic variable with two possible outcomes: (i) the molecule being photoionized or (ii) the molecule being nonionized. The corresponding probabilities are *P* and 1−P, respectively [see Eq. (1)]. Then, the standard deviation *s* of the molecular charge, according to the binomial distribution and considering that a single photoionization event predominantly leads to a charge of *Q* = 2, is $s=Q\sqrt{P(1-P)}$, agreeing reasonably well with the simulation results. A slight discrepancy occurs as there are also a few trajectories leading to charge states other than *Q* = 2 (e.g., *Q* = 1, 3, and 4). This relation is also valid for the other fluence cases very early in the pulse. However, when entering the nonlinear regime, the behavior starts to deviate from this simple picture. Moreover, at the highest fluence, an overshoot can be observed as a function of time [Fig. 3(c)]. This is a consequence of reaching saturation, i.e., at the end of the pulse, the variety of states reachable is smaller than that in the middle of the pulse. This reasoning is also supported by the fact that XMDYN-REF exhibits the overshoot feature, too. Overall, as a consequence of the facts that the low ionization case is reasonably well described analytically using the binomial distribution, and that the observed standard deviations do not exceed a few charges even at higher molecular ionization, we can also conclude that the relative standard deviation compared to the mean charge decreases as the mean value increases.

### C. Partial atomic charges

**FIG. 4.**
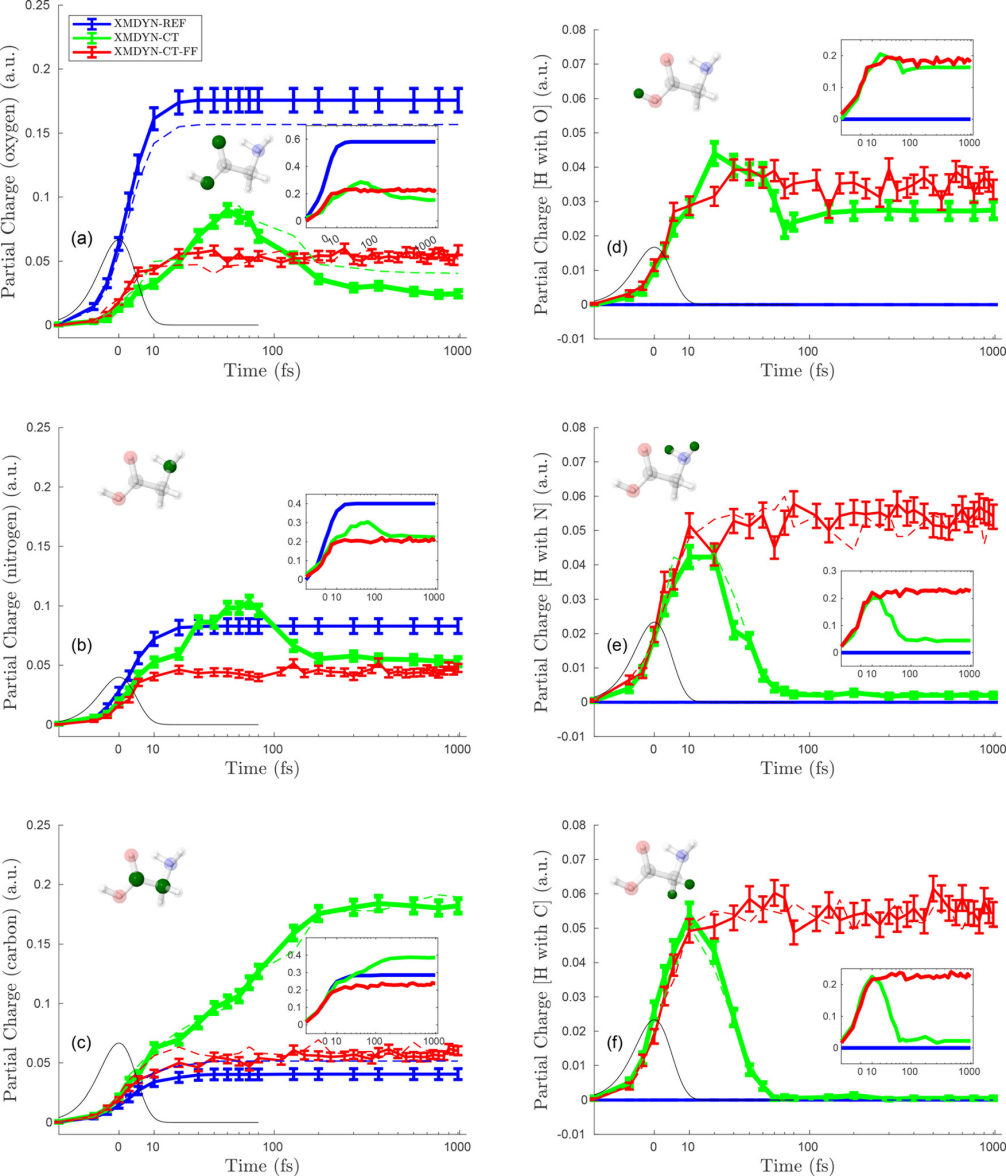
Average partial charges for the individual atoms at fluence 10^13^ photons/ $\mu m^{2}$ for three different models (blue for XMDYN-REF, green for XMDYN-CT, and red for XMDYN-CT-FF). Left panels (a)–(c) are for the non-H elements: oxygen, nitrogen, and carbon, respectively; solid lines are for the atoms with bonding to hydrogen (O_H_, N_H_, and C_H_, respectively, following the labeling in Fig. 1), while the dashed ones are for the atoms without bonding to hydrogen (labeled as O and C in Fig. 1). The panels on the right (d)–(f) are for the hydrogen atoms connected to the element presented in the panel to the left of each of them, i.e., H_O_, H_N_, and H_C_, respectively. Black lines represent the temporal envelope of the XFEL pulse intensity. The insets depict the corresponding standard deviations. The atoms analyzed in a panel are represented by solid green color in the ball-stick image.

**FIG. 5.**
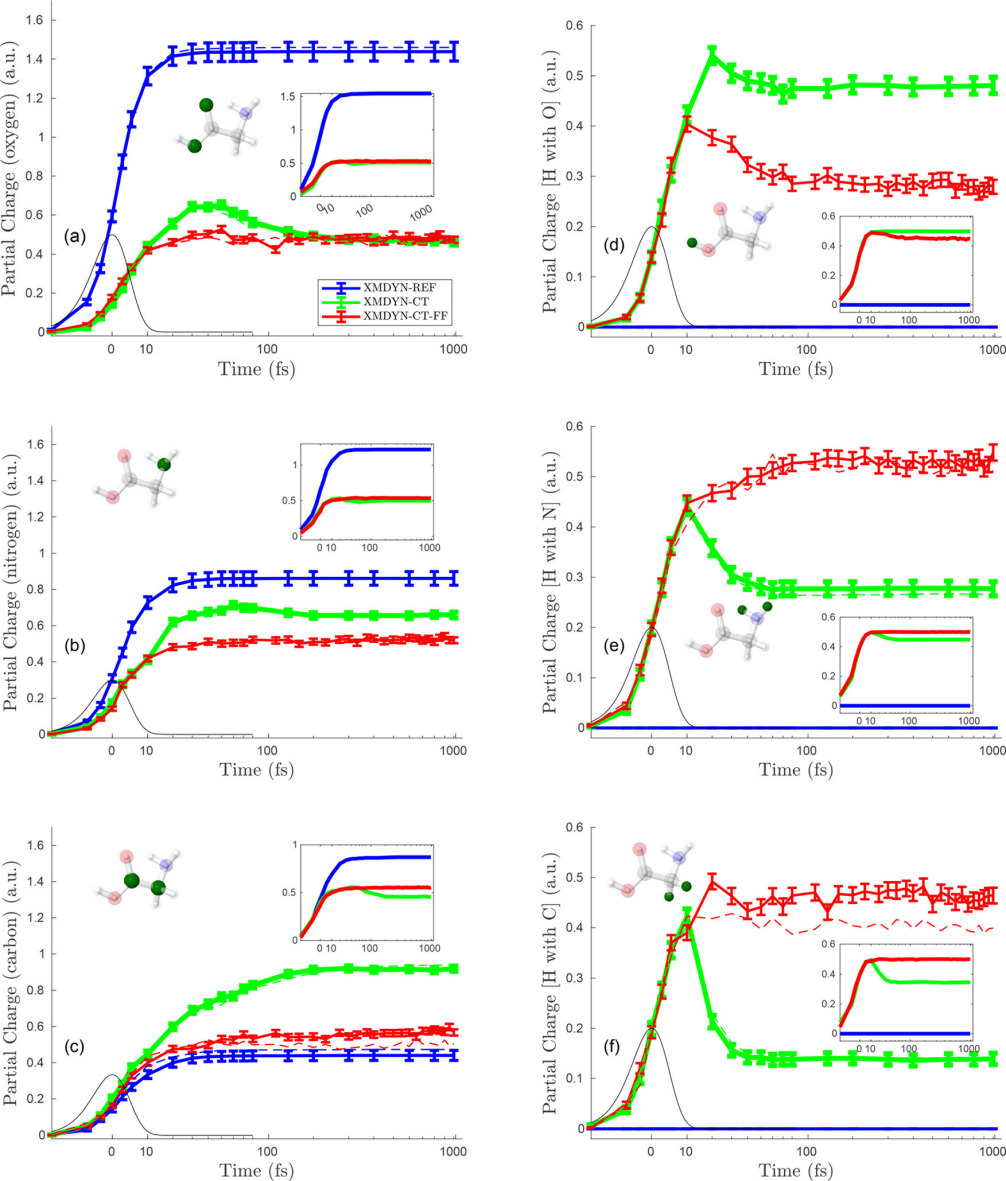
Partial charges for individual atoms at fluence 10^14^ photons/*μ*m^2^. The organization of the panels is the same as in Fig. 4.

**FIG. 6.**
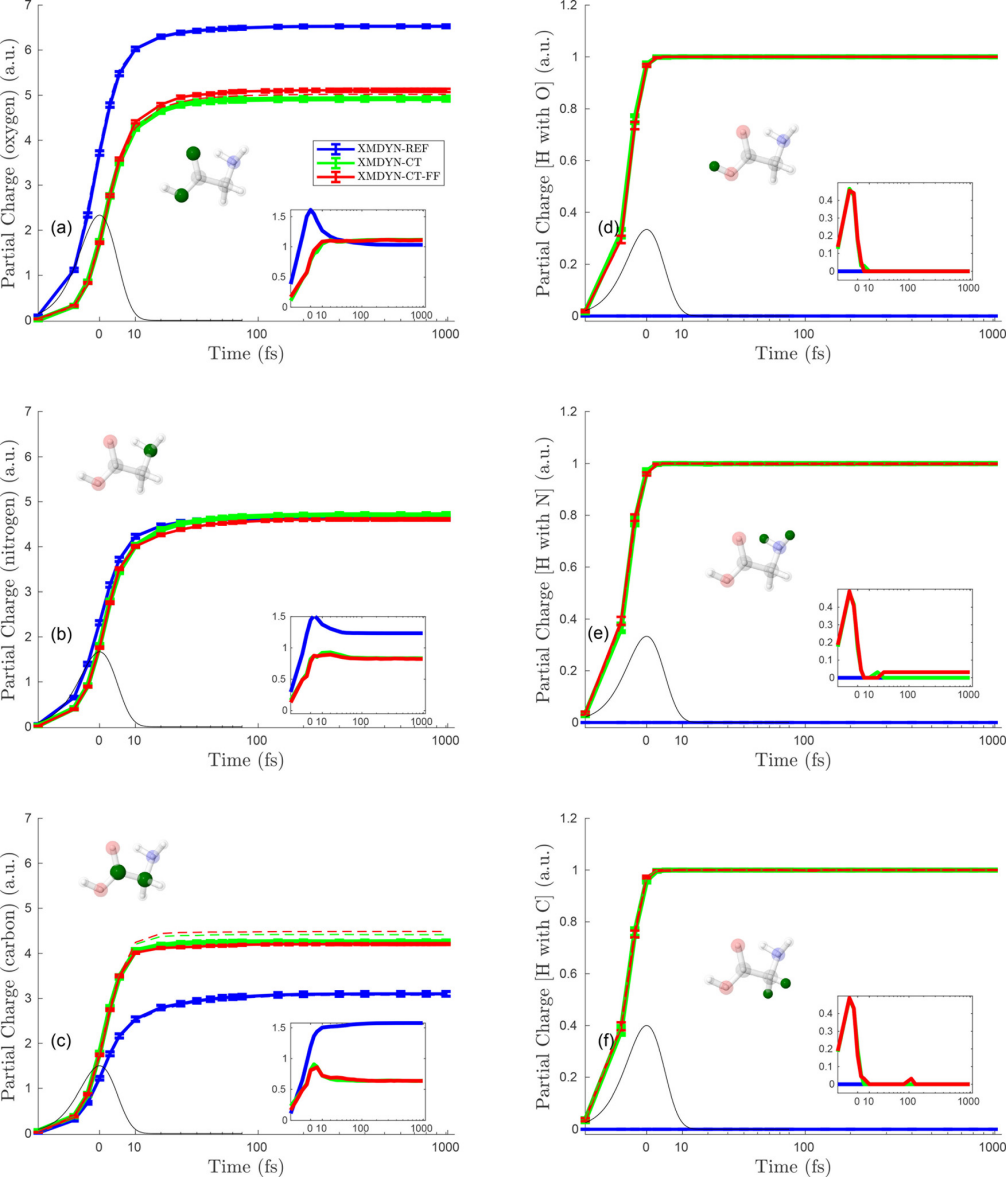
Partial charges for individual atoms at fluence 10^15^ photons/*μ*m^2^. The organization of the panels is the same as in Fig. 4.

Figures 4–6 depict the temporal evolution of the average partial charge for each individual atom in the molecule corresponding to the situations where the molecule is irradiated at fluences of 10^13^, 10^14^, and 10^15^ photons/*μ*m^2^, respectively.

We note that while for an atom that is an isolated atomic fragment, the partial charge represents the final (time independent) charge of that atom, for an atom that is part of a molecular fragment, the partial charge may still fluctuate due to charge transfer within the fragment.

Figure 4 represents the lowest fluence (10^13^ photons/*μ*m^2^) scenario. Panels on the left represent non-H atoms, whereas the ones on the right are for H atoms. Results obtained using the three different variants of XMDYN are depicted with three different colors in each panel. Note that partial charges in Fig. 4 are very small. With the independent atom treatment in XMDYN-REF, non-H atoms acquire charge depending on the element specific atomic photoionization cross section. Therefore, the final charges in panels a, c, and e show that the partial charge scales with the photoionization cross section. We also observe that the partial charge for non-H atoms increases during the initial stage, while the pulse is interacting with the molecule. This is due to the photoionization and subsequent Auger events happening around that time. However, afterward, once these processes are over, the charges reside on each individual atomic site resulting in a flat profile. Furthermore, since the ionization cross section being negligibly small for hydrogens, within XMDYN-REF, we see no ionization in the right panels of Fig. 4.

Within the XMDYN-CT framework, qualitatively different behaviors can be observed. In the beginning, the partial charge for H atoms rises along with the non-H atoms because of charge transfer. However, at a later time (around 90 fs), the charge of H_N_ and H_C_ drops to zero, while the charge of H_O_ ends up at a nonzero value. The heavier atoms exhibit nonuniform behavior, too: while the charge of O and N drops after the initial increase, the charge of C reaches its asymptotic value via a monotonic increase. The reason for this is the interplay of (full atomic) fragmentation with the phenomenon of having different threshold distances for the OTB charge transfer in the two directions between two (altogether singly charged) different atomic species as they are moving away from each other (Sec. II B). The binding energies of C, N, H, and O are 8.99, 11.49, 13.6, and 14.16 eV, respectively. These values indicate that oxygen atoms have the highest chance of keeping bound electrons during the breakup, whereas carbon atoms tend to give away their electron, in this way increasing their own charge. This is manifested even locally: the hydrogen near oxygen tends to keep its positive charge, whereas hydrogens near C or N tend to keep their valence electron during the separation from the heavy partner.

The results for XMDYN-CT-FF also show the early rise in partial charges. However, in this case, all charges stabilize at a very similar value. This is due to the fact that the Coulomb repulsion is not strong enough to breakup the molecule, and, therefore, charge transfer can go on among all atoms during the simulated 1 ps time window.

Figure 5 shows the partial charges for all individual atoms at 10^14^ photons/*μ*m^2^. Our description of Fig. 4 for the features is observed, and the respective phenomena responsible for them apply here as well, except for the following additional observations. First, the scale here for the partial charges shows higher magnitudes (within all XMDYN schemes), which is obvious since higher fluence results in more photoionization events. Second, the hydrogens bonded with C and N do not show zero final partial charge within XMDYN-CT, unlike Fig. 4. Nonetheless, the partial charges for H atoms show a trend that the H atom connected with O has more average partial charge than the one connected with N, and then least for the one connected with C. This relates again to how the different valence orbital energies lead to asymmetric charge transfer beyond critical distances between atom pairs. Third, final partial charges obtained from XMDYN-CT-FF lie above XMDYN-CT for the H atoms connected with N and C, but lie below XMDYN-CT for the H atoms connected with O. This indicates that a significant fraction of these hydrogens (bonded with O) got ejected because of higher fluence and do not take part anymore in charge transfer with O, whereas the remaining ones are still bonded with O and charge hopping works for them. Therefore, the average partial charge goes below the one obtained in XMDYN-CT.

Figure 6 shows the partial charge for each individual atom corresponding to the fluence of 10^15^ photons/*μ*m^2^. Because of the high fluence, the charges in each panel show higher values as compared to Figs. 4 and 5. As the discussion on Fig. 4 already covers the common features observed also in this case, we focus on additional features appearing only at such a high fluence. First, when using XMDYN-CT, all atoms reach their final charge state at a very early stage (∼10 fs); this is because the strong Coulomb repulsion due to the high charges on each atom makes them rapidly move away from each other. Second, the results corresponding to XMDYN-CT-FF follow the same profile as XMDYN-CT, reflecting the fact that chemical bonds do not play a significant role at such high fluence.

Finally, we note that in the fluence regime investigated, the standard deviation of partial charges shows a very similar behavior to that of the total molecular charge, as a function of fluence. The only exception is the standard deviation for the hydrogen atoms by the end of the pulse: at the highest fluence considered, due to the mean atomic charge being well above one, their charge saturates, leading to vanishing standard deviation.

### D. Average displacement

**FIG. 7.**
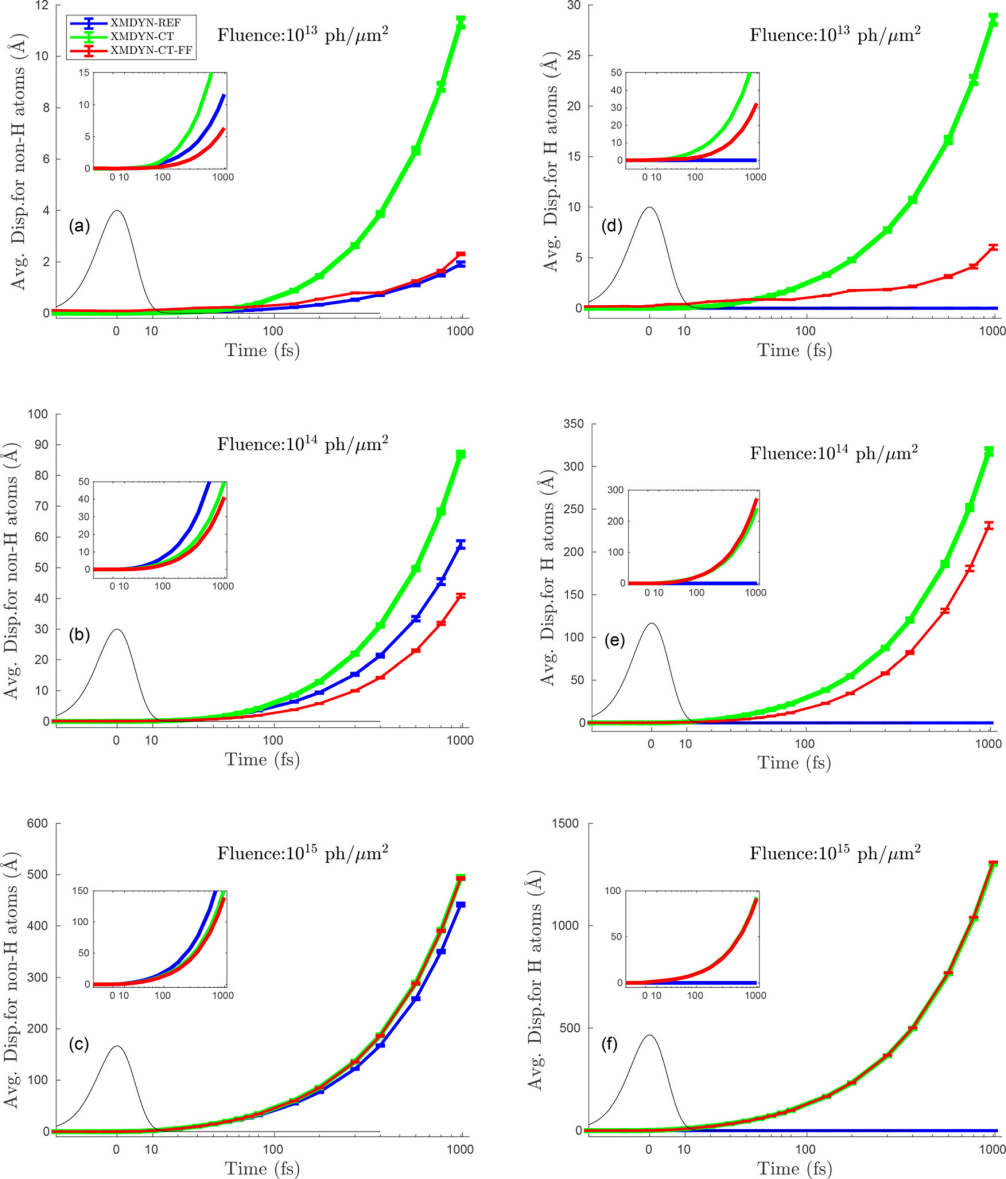
The panels on the left correspond to the average displacement of non-H atoms, whereas the panels on the right depict the same for the hydrogens. The top two panels depict the scenario for a fluence of 10^13^ photons/*μ*m^2^, whereas the two in the middle correspond to 10^14^ photons/*μ*m^2^, and the bottom panels are for 10^15^ photons/*μ*m^2^. Each of the six panels shows the results obtained from three different versions of XMDYN (indicated using three different colors) and the standard deviations corresponding to the respective dataset are shown in the inset of each panel.

The time-dependent, realization-averaged atomic displacements are depicted in Fig. 7. The first, general observation is that in the case of XMDYN-REF (blue lines), the mean displacement of hydrogen atoms at all fluences is practically zero, due to the suppression of ionization. Furthermore, the trends in the displacement of H atoms for XMDYN-CT and XMDYN-CT-FF are very similar to that of the non-H atoms for all three fluences, only exhibiting larger values. This is due to the fact that hydrogen is an order of magnitude lighter than non-H atoms, leading to faster movement when experiencing the same forces.

At the lowest fluence investigated, the molecules in the majority of the trajectories stay nonionized; only in ∼23% of the trajectories, the system becomes ionized due to a single photoionization event, and the probability of multiple photoionization is just a few percent. When using XMDYN-REF, the single photoionization event typically leads to a doubly charged molecule, but the charge remains localized to a single non-H atom. Therefore, in the vast majority of the trajectories, no Coulomb repulsion appears. This yields only a small average displacement, which is due to the recoil momentum the ionized atom acquires at electron ejection [Figs. 7(a) and 7(d), blue lines]. In XMDYN-CT and XMDYN-CT-FF, charge transfer leads to ongoing redistribution of charges, transiently charging typically two atoms at the same time in most of the ionized trajectories. This leads to Coulomb repulsion between the charged atoms at a given time. Therefore, in XMDYN-CT, an increased average displacement can be observed. However, in XMDYN-CT-FF, the displacements are highly suppressed due to the chemical bonds acting against Coulomb repulsion. We also note that certain irregularities can be observed in XMDYN-CT-FF results. This is due to the fact that in all trajectories, the same initial atomic coordinates (away from the energy minimum of the force field) were used, which creates a correlation/coherence among slightly ionized trajectories (nonionized trajectories exhibit exactly the same atomic movements).

When increasing the fluence, an increasing number of atoms possess charge after irradiation. Still, the same trends can be observed. At the same time, the importance of bonds vanishes: at the highest fluence, XMDYN-CT and XMDYN-CT-FF lead to practically the same behavior, and even XMDYN-REF is very similar to the previous two.

It is worthwhile to note that the standard deviations in all the panels are quite large. The relative fluctuation is really high especially for 10^13^ and 10^14^ photons/*μ*m^2^: the small total charge leads to a more inhomogeneous charge distribution among the atoms when comparing the individual realizations to each other (indicated also by the larger relative charge fluctuations, discussed in Sec. III B). This ultimately leads to more diverse scenarios of the atomic movements. On the other hand, when all the atoms are ionized to a large degree, i.e., at the highest fluence (i.e., 10^15^ photons/*μ*m^2^), a relatively more homogeneous charge redistribution leads to smaller relative standard deviation.

Furthermore, in the course of analysis, we also extract the time (relative to the peak of the pulse) at which the average displacement corresponding to the non-H atoms reaches a value of 1 Å (Table I). This information is useful from the perspective of single-particle imaging applying the considered irradiation conditions because it provides an idea whether the molecule retains its structural form on the scale of atomic resolution, or whether the molecule undergoes significant structural damage already during the pulse. We notice that the average displacement reaches 1 Å well beyond the temporal width of the pulse for fluences 10^13^ and 10^14^ photons/*μ*m^2^, whereas this critical time lies very close to the temporal width (even within the pulse width for XMDYN-REF) for 10^15^ photons/*μ*m^2^.

**TABLE I.** Critical times after the center of the pulse at which the average displacement of the non-H atoms reaches 1 Å.

Model	10^13^ ph/*μ*m^2^ (fs)	10^14^ ph/*μ*m^2^ (fs)	10^15^ ph/*μ*m^2^ (fs)
XMDYN-REF	89.97	17.77	3.66
XMDYN-CT	66.15	24.05	5.01
XMDYN-CT-FF	142.44	31.71	5.40

### E. Discussion

Based on the analysis presented, we conclude that chemical phenomena, i.e., charge redistribution and bonds, play an important role in the dynamics of small organic molecules comprising light elements for presently experimentally achievable fluences. The critical fluence above which chemical phenomena, in particular bonds, are irrelevant in the dynamics of such small molecules is far beyond our access at present. However, we also point out that, as our findings predominantly depend on the total charge acquired, in two classes of systems, the critical fluence is expected to lie at a lower value. The first is when the molecule contains a heavy atom, which enhances ionization. The second is the case of extended systems, when collisional ionization yields higher charge states of the constituent atoms, leading to stronger intramolecular Coulomb repulsion. Therefore, this study is a step toward finding the fluence regime where chemical bonds become insignificant in the dynamics of arbitrary molecules irradiated under imaging conditions.

Furthermore, we also note that the actual number of atomic bound electrons (directly connected to the charge states of the atoms analyzed in our study) is crucial in single-particle imaging when identifying and differentiating the elements. Therefore, fluctuation of the partial charges implies fluctuation in the individual scattering patterns obtained during single-molecule measurement that may affect the structure reconstruction procedure.

We point out again that in the current approach, we disregard any change of the bonds due to the nonzero charge state of the molecule during the dynamics, using a force fields corresponding to neutral glycine in its electronic ground state. In reality bond softening, bond breaking occurs during ionization. Therefore, the real molecular behavior of the system may be expected within the region enclosed by the results of XMDYN-CT (no bonding) and XMDYN-CT-FF (too much bonding).

It is important to note that CREXIM is observed in XMDYN simulations (when the phenomenon of charge transfer is included), which suggests that an expensive, full quantum-mechanical calculation is not necessarily required to capture the effect. However, it is still an open question to what degree quantitative agreement holds between the results of *ab initio* calculations and the hybrid modeling employed here.

It is worthwhile to mention that simulating x-ray-irradiated samples with a size much larger than that of glycine is feasible. This was demonstrated in previous studies using XMDYN, e.g., on nitrogenase iron protein molecules (containing ∼5000 non-hydrogen atoms)^21^ and on 50 000-atom-size Krypton clusters.^42^ Moreover, incorporating the Barnes-Hut treecode algorithm in XMDYN has recently shown significant improvement to the computational efficiency.^43^ However, in these studies, the currently used ReaxFF module was not included. In the present calculation, no optimization of the interfacing between XMDYN and ReaxFF was performed. Hence, we observed for a realization an increase in computational time from approximately 3 min (XMDYN without ReaxFF) to 3 h (XMDYN combined with ReaxFF). While this did not thwart the simulations of glycine molecules for the current study, simulations of significantly larger systems will require substantial optimization and future developments.

## IV. CONCLUSIONS

In this paper, we reported on a theoretical study focusing on the effect of charge transfer and chemical bonds on the dynamics of small biological molecules irradiated by a single XFEL pulse. We achieved this by using three model variants of the molecular dynamics based classical-quantum hybrid computational framework XMDYN. The chemical bonds were approximated by the reactive force field (ReaxFF) description, independent of the ionization state of the molecule in order to maximize the effect of bonds. We analyzed in detail the features observed in key quantities of the dynamics, i.e., in the time dependent charge states and atomic displacements, occurring at a variety of fluences. At high fluence, due to the high charge state reached, bondless simulation yields accurate results. However, chemical phenomena are important at fluences realistically achievable experimentally today. We point out that our conclusions are relevant primarily for small organic molecules. For systems containing a significant number of heavy elements or that are of larger size, the limiting fluence beyond which bondless modeling is already accurate is expected to be smaller. In this work, we also demonstrated that XMDYN is capable of capturing the phenomenon of charge-rearrangement-enhanced x-ray ionization of molecules (CREXIM), observable at high ionization degree.

## Data Availability

The data that support the findings of this study are available from the corresponding authors upon reasonable request.
